# Commentary: Increased Beat-to-Beat Variability of T-Wave Heterogeneity Measured From Standard 12-Lead Electrocardiogram Is Associated With Sudden Cardiac Death: A Case-Control Study

**DOI:** 10.3389/fphys.2020.598314

**Published:** 2020-10-19

**Authors:** Vijay S. Chauhan, Juan Pablo Martínez, Marcel A. G. van der Heyden

**Affiliations:** ^1^Division of Cardiology, Peter Munk Cardiac Centre, University Health Network, Toronto, ON, Canada; ^2^Aragón Institute of Engineering Research, IIS Aragón, Universidad de Zaragoza, Zaragoza, Spain; ^3^Biomedical Research Networking Center in Bioengineering, Biomaterials, and Nanomedicine (CIBER-BBN), Zaragoza, Spain; ^4^Division of Heart & Lungs, Department of Medical Physiology, University Medical Center Utrecht, Utrecht, Netherlands

**Keywords:** electrocardiagram (ECG), T-wave, sudden cardiac death (SCD), risk markers, coronary artery disease, arrhythmia

The electrocardiogram (ECG) reflects the electrical activity within the heart. Following the discovery of the small electrical signals in the human heart, the Dutch scientist Willem Einthoven developed sensitive methods for detecting them and recognized their clinical implications (Kligfield, 2002). For his contributions Willem Einthoven, whose scientific roots originated from the Utrecht physiology department (Einthoven, 1885), was awarded the Nobel Prize in 1924. Since then, many enigmas of the ECG have been solved and its intricate information carries valuable clues for clinical decision making.

*In vivo* mapping studies have established that spatial heterogeneity in repolarization is a requisite for re-entrant ventricular arrhythmias by effecting unidirectional conduction block. Important modulators of spatial heterogeneity of repolarization include autonomic tone, ischemia, heart rate and premature or ectopic beats, which can produce temporal heterogeneity in the order of seconds, minutes, or hours depending on their time-constants. In the contemporary era of dynamic surface electrocardiography, a formidable challenge is quantifying spatiotemporal repolarization heterogeneity with sufficient fidelity to image the arrhythmogenic myocardial substrate and thereby provide indices for a patient's risk of arrhythmic death (Laguna et al., 2016).

Hekkanen et al. (2020) performed a large case-control study of 200 victims of sudden cardiac death (SCD) due to autopsy-proven coronary artery disease, who were age-, and sex-matched to controls with angiographically-confirmed coronary artery disease from the ARTEMIS database. A comprehensive analysis of their 12-lead ECGs within a mean of 1.8 months from SCD was performed to evaluate several measures of spatial repolarization heterogeneity, including an assessment of their temporal variability based on the standard deviation (SD) of each measure over 5 consecutive beats. The major findings were that T wave heterogeneity (TWH), TWH-SD, and QT-SD independently discriminated cases from controls in Cox multivariate regression analysis [adjusted Hazard ratio 1.05 (95th confidence interval 1.02–1.09)] after adjusting for baseline differences in beta-blocker use and heart rate. Although cases consistently had higher temporal variability in their repolarization metrics, this was unrelated to their higher resting heart rate or lower beta-blocker usage. The strength of this study is its large sample size of 12-lead ECGs from accurately phenotyped cases of SCD. The assessment of repolarization metrics and their short-term temporal variability from a routine test such as the standard 10 s ECG in sinus rhythm is also comprehensive and novel. Such metrics, if proven to be prognostic in broader populations, would open the door to large-scale screening for SCD risk.

However, an important consideration when assessing the proposed short-term metrics is the effect of recording noise and noise-control (Waks et al., 2015). ECGs were digital and the analysis of repolarization metrics were automated, but no details were provided regarding ECG quality or any pre-processing to improve signal-to-noise ratio. Nearing and Verrier (2003) first described TWH to track interlead heterogeneity of R- and T wave morphology in a resting 12-lead ECG. Their assessment required a median QRS-T complex from each 10 s precordial lead and then computation of the splay or heterogeneity about this median axis. In a 5600 subject cohort study with 12-lead ECGs, TWH in the left precordial leads (V4–V6) independently predicted SCD [adjusted Hazard ratio 1.4 (95th confidence interval 1.2–1.8) per increment of one standard deviation] (Kenttä et al., 2016). In contrast, Hekkanen et al. (2020) computed TWH from a single beat in order to derive TWH-SD from 5 consecutive beats. This approach necessitates high quality beat-to-beat ECG recordings and there was no assessment of noise or reproducibility by the authors. Also, the standard error of the standard deviation estimation from a noisy series depends on the noise level and the number of samples considered for the estimation. Thus, it should be established how noise present in the ECG affects the TWH-SD metric, in order to define the required ECG quality or the minimum number of beats necessary for a reliable estimation of the physiological variability. In the end, the prognostic utility of TWH-SD was only modest and similar to TWH based on their adjusted Hazard ratios, which was less than that reported by Kenttä et al. (2016) in a lower risk general Finish population.

Another consideration potentially affecting reliability of the proposed indices is physiologic reproducibility of the short-term variability of ventricular repolarization heterogeneity. How stable can it be if it is affected by other known modulating factors, such as circadian variation, heart rate or autonomic tone? Rizas et al. (2014) elegantly demonstrated low-frequency rhythmic modulations of repolarization associated with changing sympathetic activity during steady-state ventilation, passive head-up tilt testing, low-intensity exercise testing and beta-blockade. Low-frequency repolarization oscillations, so-called periodic repolarization dynamics (PRD), were assessed from a 12-lead ECG's beat-to-beat changes in T-wave spatial orientation in 5-min segments. PDR was a strong, independent predictor of SCD in a large retrospective and prospective cohort of post-infarction patients with and without reduced left ventricular ejection fraction (Rizas et al., 2017, 2019), suggesting that abnormal spatiotemporal repolarization or its short-term sympathetic modulation may ultimately provide the substrate for lethal ventricular arrhythmias. In light of this work, it is unexpected that TWH-SD and QT-SD were similar between subjects on or off beta-blockers whether SCD cases or controls, but this analysis may be underpowered.

In summary, the development of novel 12-lead ECG-based metrics of beat-to-beat spatiotemporal repolarization heterogeneity, such as TWH-SD and QT-SD may ultimately improve the assessment of SCD risk and better inform patient selection for prophylactic defibrillator therapy. However, important challenges remain, including defining the quality requirements for ECG recordings, improving short-term reproducibility, and controlling for heart rate and autonomic tone as potential confounders. Further studies are also needed to understand how sudden beat-to-beat changes in T wave morphology across ECG leads reflect intracardiac repolarization heterogeneity during acute physiologic stresses that can trigger SCD. Inherent to these studies will be the need for simultaneous, non-invasive monitoring of autonomic tone (i.e., heart rate, blood pressure, respiration) to better understand the short-term regulatory control of ventricular repolarization, which may vary depending on the underlying inherited or acquired cardiovascular disease.

## Author Contributions

VC, JM, and MH conceptualized, wrote, and revised the manuscript. All authors reviewed and agreed upon the final manuscript.

## Conflict of Interest

The authors declare that the research was conducted in the absence of any commercial or financial relationships that could be construed as a potential conflict of interest.

## References

[B1] Einthoven W. (1885). Stereoscopie door kleurverschil [Ph.D. thesis]. Utrecht University, Utrecht, Netherlands.

[B2] Hekkanen J. J. Kenttä T. V. Haukilahti M. A. E. Rahola J. T. Holmström L. T. A. Vähätalo J. (2020). Increased beat-to-beat variability of t-wave heterogeneity measured from standard 12-lead electrocardiogram is associated with sudden cardiac death: a case-control study. Front. Physiol. 11:1045 10.3389/fphys.2020.01045 32982784 PMC7477294

[B3] Kenttä T. V. Nearing B. D. Porthan K. Tikkanen J. T. Viitasalo M. Nieminen M. S.. (2016). Prediction of sudden cardiac death with automated high-throughput analysis of heterogeneity in standard resting 12-lead electrocardiograms. Heart Rhythm 13, 713–720. 10.1016/j.hrthm.2015.11.035 26616400

[B4] Kligfield P. (2002). The centennial of the Einthoven electrocardiogram. J. Electrocardiol. 35, 123–129. 10.1054/jelc.2002.37169 12539109

[B5] Laguna P. Martínez J. P. Pueyo E. (2016). Techniques for ventricular repolarization instability assessment from the ECG. Proc. IEEE 104, 392–415. 10.1109/JPROC.2015.2500501

[B6] Nearing B. D. Verrier R. L. (2003). Tracking cardiac electrical instability by computing interlead heterogeneity of T-wave morphology. J. Appl. Physiol. 95, 2265–2272. 10.1152/japplphysiol.00623.2003 12897035

[B7] Rizas K. D. Doller A. J. Hamm W. Vdovin N. Von Stuelpnagel L. Zuern C. S.. (2019). Periodic repolarization dynamics as a risk predictor after myocardial infarction: prospective validation study. Heart Rhythm 16, 1223–1231. 10.1016/j.hrthm.2019.02.024 30818092

[B8] Rizas K. D. McNitt S. Hamm W. Massberg S. Kääb S. Zareba W.. (2017). Prediction of sudden and non-sudden cardiac death in post-infarction patients with reduced left ventricular ejection fraction by periodic repolarization dynamics: MADIT-II substudy. Eur. Heart J. 38, 2110–2118. 10.1093/eurheartj/ehx161 28431133 PMC5837472

[B9] Rizas K. D. Nieminen T. Barthel P. Zurn C. S. Kahonen M. Viik J.. (2014). Sympathetic activity-associated periodic repolarization dynamics predict mortality following myocardial infarction. J. Clin. Invest. 124, 1770–1780. 10.1172/JCI70085 24642467 PMC3973112

[B10] Waks J. W. Soliman E. Z. Henrikson C. A. Sotoodehnia N. Han L. Agarwal S. K.. (2015). Beat-to-beat spatiotemporal variability in the T vector is associated with sudden cardiac death in participants without left ventricular hypertrophy: the Atherosclerosis Risk in Communities (ARIC) Study. J. Am. Heart Assoc. 4:e001357. 10.1161/JAHA.114.001357 25600143 PMC4330061

